# The potential use of artificial intelligence for venous thromboembolism prophylaxis and management: clinician and healthcare informatician perspectives

**DOI:** 10.1038/s41598-024-62535-9

**Published:** 2024-05-26

**Authors:** Barbara D. Lam, Laura E. Dodge, Sabrina Zerbey, William Robertson, Rachel P. Rosovsky, Leslie Lake, Siddhant Datta, Pavania Elavakanar, Alys Adamski, Nimia Reyes, Karon Abe, Ioannis S. Vlachos, Jeffrey I. Zwicker, Rushad Patell

**Affiliations:** 1 Division of Hematology, Department of Medicine, Beth Israel Deaconess Medical Center, Harvard Medical School, 330 Brookline Avenue, Boston, MA 02215 USA; 2 Department of Obstetrics and Gynecology, Beth Israel Deaconess Medical Center, Harvard Medical School, Boston, MA USA; 3 Harvard T.H. Chan School of Public Health, Boston, MA USA; 4 Weber State University, Ogden, UT USA; 5 National Blood Clot Alliance, Philadelphia, PA USA; 6 Division of Hematology, Department of Medicine, Massachusetts General Hospital, Harvard Medical School, Boston, MA USA; 7 Division of Hospital Medicine, Department of Medicine, Beth Israel Deaconess Medical Center, Harvard Medical School, Boston, MA USA; 8 Division of Blood Disorders, National Center on Birth Defects and Developmental Disabilities, Centers for Disease Control and Prevention, Atlanta, GA USA; 9 Department of Pathology, Cancer Research Institute, Beth Israel Deaconess Medical Center, Harvard Medical School, Boston, MA USA; 10 Division of Hematology, Department of Medicine, Memorial Sloan Kettering Cancer Center, New York, NY USA; 11 Division of Clinical Informatics, Department of Medicine, Beth Israel Deaconess Medical Center, Boston, USA

**Keywords:** Venous thromboembolism, Artificial intelligence, Machine learning, Survey, Computational biology and bioinformatics, Health care, Medical research

## Abstract

Venous thromboembolism (VTE) is the leading cause of preventable death in hospitalized patients. Artificial intelligence (AI) and machine learning (ML) can support guidelines recommending an individualized approach to risk assessment and prophylaxis. We conducted electronic surveys asking clinician and healthcare informaticians about their perspectives on AI/ML for VTE prevention and management. Of 101 respondents to the informatician survey, most were 40 years or older, male, clinicians and data scientists, and had performed research on AI/ML. Of the 607 US-based respondents to the clinician survey, most were 40 years or younger, female, physicians, and had never used AI to inform clinical practice. Most informaticians agreed that AI/ML can be used to manage VTE (56.0%). Over one-third were concerned that clinicians would not use the technology (38.9%), but the majority of clinicians believed that AI/ML probably or definitely can help with VTE prevention (70.1%). The most common concern in both groups was a perceived lack of transparency (informaticians 54.4%; clinicians 25.4%). These two surveys revealed that key stakeholders are interested in AI/ML for VTE prevention and management, and identified potential barriers to address prior to implementation.

## Introduction

Venous thromboembolism (VTE) is the leading cause of preventable death in hospitalized patients^1,2^. This has long been recognized as a major public health challenge, with multiple organizations calling for increased awareness and thromboprophylaxis of patients^3–5^. Thromboprophylaxis is known to be safe and effective^6^, but numerous studies have shown that it is still misused or underused^6–9^.The first step in appropriate VTE prophylaxis involves identifying patients at risk for VTE. Prior guidelines recognized the burden of individual risk assessment and recommended a cohort-based approach to VTE prophylaxis^5^. However, these types of universal strategies have shown minimal impact on reducing VTE rates^10,11^. Contemporary guidelines emphasize the heterogeneity of hospitalized medical patients and recommend an individualized approach to risk assessment and prophylaxis^12^.

Multiple risk assessment models (RAMs) have been developed over the years to assist clinicians in risk stratifying patients. These RAMs are effective tools for identifying at-risk patients^13^, and some studies have shown that incorporating a RAM into the clinical workflow can reduce VTE events^14,15^. However, there are dozens of validated RAMs with variable risk predictors, making it difficult to determine which RAM a clinician should choose, and in which populations. An ideal RAM would be applicable to diverse patients and recognize that the risk of VTE can change over the course of a hospitalization. The RAM would be easy to use and consider both the risk of bleeding and clotting. These lofty goals make new technologies such as artificial intelligence (AI) and its subbranch, machine learning (ML), particularly promising in the field of VTE prevention. AI/ML is an area of computer science that trains computer algorithms to make predictions based on available data. An effective AI/ML tool would search the medical chart for relevant risk factors, weigh the risk of VTE as well as the risk of bleeding, alert the clinician when that risk changes, and assist the clinician in making a decision about appropriate VTE prophylaxis.

A recent systematic review and meta-analysis of AI/ML tools for VTE prevention showed overall high performance^16^, but few AI/ML-based models have been implemented in clinical settings^17^. More research is needed to understand the safe and effective implementation of AI/ML ^17^. Understanding stakeholder attitudes is an important part of the path to implementation. In this paper, we present two surveys, one directed at clinicians and the other at informaticians, to better understand their views on AI/ML in the field of VTE prevention and management.

## Methods

### Survey design

We conducted two cross-sectional electronic surveys about perceptions on the use of AI for VTE prevention among clinicians (Supplementary data 1) and among healthcare informaticians (Supplementary data 2). Both study instruments were designed and administered in REDCap, a secure web-based application^18^. Both were vetted by a multidisciplinary team with expertise in hematology, informatics, and public health, and refined for clarity, length, and relevance with a survey expert. They were individually approved by the Beth Israel Deaconess Medical Center IRB as exempt protocols and all research was performed in accordance with relevant guidelines and regulations. Informed consent was obtained at the start of each survey via a statement about the voluntary nature of the study.

We defined VTE as deep vein thrombosis (DVT) and/or pulmonary embolism (PE). We defined prophylaxis as including pharmacologic and/or mechanical measures for VTE prevention, and we used the terms prophylaxis and prevention interchangeably. In the healthcare informatician survey, we used the term “blood clot” instead of VTE because not all respondents may be familiar with the term VTE. We defined AI/ML as technologies that mimic human intelligence, and gave an example of technology using a large dataset to train a model that can predict outcomes or help make decisions. We used the terms AI and ML interchangeably.

The clinician survey explored attitudes and practices around VTE prevention, with a specific section exploring the use of AI/ML for VTE prevention (3 questions). We report the findings of that section here. The healthcare informatician survey explored attitudes and practices around AI/ML in healthcare in general, with a specific section on the use of AI/ML for VTE prevention (21 questions total). We report the findings of the survey in its entirety here.

Survey items included both quantitative and qualitative questions. Quantitative questions used Likert scales and Yes/No/Do not know responses. Several questions provided respondents with a field to write a free-text response. Both surveys asked respondents about general demographics such as age, sex, ethnicity, role, and time in practice (Supplementary data 1 and 2).

### Respondents

The surveys were administered to targeted convenience samples between August 2021 and December 2022. Clinical respondents were recruited through social media, professional organizations, and program directors across the United States (US). Clinical trainees could opt to enter a raffle drawing for one of 100 $10 gift cards. There was no incentive provided for other groups. Healthcare informaticians were recruited through professional organizations and a publicly available database listing recipients of National Institutes of Health informatics grant awardees. Due to these methods of recruitment, a response rate could not be accurately calculated. As a descriptive survey utilizing a sample of convenience, we did not perform any a priori sample size calculations.

### Analysis

We used descriptive statistics to present responses and respondent characteristics, summarizing data as proportions. Binomial 95% confidence intervals (CI) was included for primary outcomes. We used the chi square test to compare categorical responses between groups. We performed all analyses using SAS, version 9.4 (SAS Institute, Cary, NC, USA).

All clinicians were asked if AI/ML can help with VTE prevention in hospitalized patients; respondents who answered anything other than “definitely yes” were asked what concerns they have about AI/ML and provided a list of options derived from the literature, along with space to write a free-text response. Two researchers (BDL and RP) conducted a qualitative analysis of these answers to batch responses that matched existing categories and to categorize new themes derived from the data. All conflicts were settled by consensus.

All healthcare informaticians were asked if AI/ML can help with management of blood clots. Respondents who answered yes were asked what aspects of blood clot management AI/ML could be useful for. All respondents were asked about potential barriers and what else to consider when using AI/ML to assist with clinical management of blood clots. Two researchers (BDL and RP) conducted a qualitative analysis of these answers. All qualitative analysis for both surveys was done by reviewing responses, developing a codebook, and using the codebook to independently code all free text responses. Themes were derived from the data. Up to two codes were assigned to each response, and the researchers compared their codes and reached consensus for every response. All qualitative coding was completed with Microsoft Excel (Microsoft Corp., Seattle, WA, USA).

## Results

### Healthcare informatician—respondent characteristics

Of 101 respondents to the healthcare informatician survey, a majority were greater than 40 years old (54.5%), male (62.0%), and white (70.1%) (Table 1). Respondents could identify as more than one role; most were clinicians (44.6%), followed by data scientists (36.6%). Most respondents reported that they had been practicing in informatics for more than 10 years (54.5%).

**Table 1:** Healthcare informatician demographic characteristics.

Characteristic	N = 101, n (%)
Age	
≤ 40 years	45 (45.5%)
> 40 years	54 (54.5%)
Gender	
Female	38 (38.0%)
Race/ethnicity^a^	
Asian	19 (19.6%)
Black or African American	7 (7.2%)
White	68 (70.1%)
Other	4 (4.1%)
Hispanic or Latino	8 (8.1%)
Role^a^	
Clinician	45 (44.6%)
Data scientist	37 (36.6%)
Clinical informaticist	30 (29.7%)
Biomedical informaticist/computational biologist	29 (28.7%)
Chief Medical Information Officer (CMIO) or other medical informatics officer	5 (5.0%)
How long have you been practicing in informatics?	
≤ 10 years	31 (31.3%)
> 10 years	54 (54.5%)

Not all participants answered these optional questions, so the denominator may vary.

^a^Respondents could select more than one, thus totals may sum to greater than 100%.

Most felt they were very well informed (41.6%) or sufficiently informed (48.5%) about AI/ML, with a majority reporting that they had taken coursework on the topic (61.4%) or were doing research on the topic (68.3%). A large portion also reported that they had worked on deploying AI/ML (42.6%).

### Clinician survey—respondent characteristics

Of 607 US-based respondents to the clinician survey, a majority were 40 years old or younger (69.9%), female (55.7%), and white (68.2%) (Table 2). Physicians made up 70.7% of respondents, of which 45.4% were trainees. Hospital medicine was the most common specialty (52.1%) followed by hematology (20.8%). A majority of respondents (65.8%) reported making a decision about whether a patient needs VTE prophylaxis every day. Only 20.5% of respondents reported that they had used AI/ML to inform their clinical practice; a majority had not (57.9%) or were unsure (21.6%).

**Table 2:** Clinician demographic characteristics.

Characteristics	N = 607, n (%)
Age	
≤ 40 years	423 (69.9%)
> 40 years	182 (30.1%)
Gender	
Female	335 (55.7%)
Race/ethnicity^a^	
Asian	124 (20.4%)
Black or African American	22 (3.6%)
White	414 (68.2%)
Other	45 (7.4%)
Hispanic or Latino	45 (7.4%)
Clinical role	
MD or DO	429 (70.7%)
Pharmacist	125 (20.6%)
Registered nurse	25 (4.1%)
Advanced practice provider (APP) specified as nurse practitioner or physician assistant	27 (4.4%)
MD, DO, or APP time in clinical practice (n = 456)	
In training	207 (45.4%)
Not in training	237 (52.0%)
Completed training, practicing 10 years or fewer	121 (51.1%)
Completed training, practicing more than 10 years	116 (49.0%)
Unknown training status	12 (2.6%)
Clinical focus^a^	
Hospital medicine	316 (52.1%)
Hematology	126 (20.8%)
Primary care	89 (14.7%)
Critical care	76 (12.5%)
Cardiology or vascular medicine	55 (9.1%)
Other	141 (23.2%)
Primary practice setting	
Academic hospital	383 (63.1%)
Community hospital	211 (34.8%)
VA hospital or other	13 (2.1%)

Not all participants answered these optional questions, so the denominator may vary.

^a^Respondents could select more than one, thus totals may sum to greater than 100%.

### Healthcare informatician—experiences with AI/ML

Most informaticians (62.6%) reported that their organization is using or developing AI/ML for healthcare. Of these 62 respondents, a majority described the status of AI/ML at their organization as implemented with at least one model in use (82.3%), and that their organizations primarily develop the models themselves (81.4%). Less than half (45.8%) reported using third-party vendors or partnering with local universities (28.8%).

Respondents who reported developing AI/ML systems used Python (76.6%), R (45.3%), and toolkits (42.2%). Of the respondents who described what toolkit they prefer, the most commonly cited ones were Scikit-learn and TensorFlow.

### Healthcare informatician—attitudes towards AI/ML

**Figure 1:**
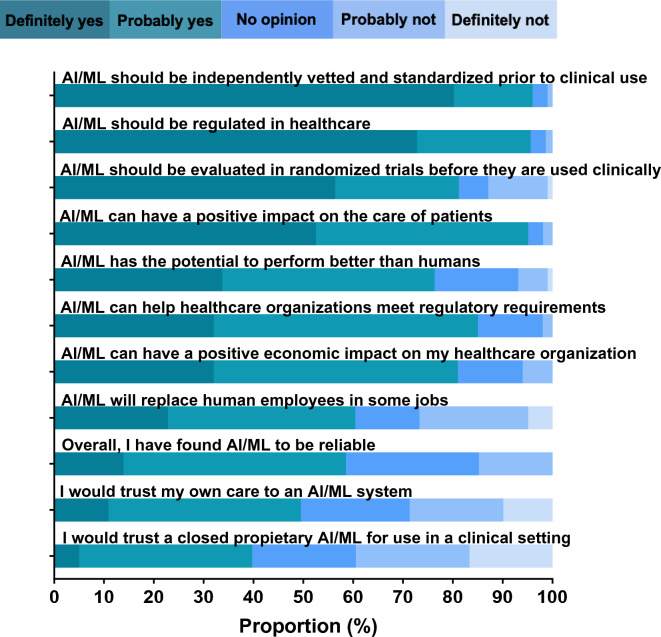
Informatician attitudes towards AI/ML (n = 101).

A majority of informaticians agreed that AI/ML can have a positive impact on the care of patients (95.0%), can help healthcare organizations meet regulatory requirements (95.0%), and can have a positive economic impact on their healthcare organization (81.0%) (Fig. 1, Supplementary Table 1). Informaticians mostly agreed that AI/ML has the potential to perform better than humans (76.3%) and will replace human employees in some jobs (60.4%). Respondents found AI/ML to be overall reliable (58.5%) and would trust their own care to an AI/ML system (49.5%). However, less than half would trust a closed proprietary system (39.7%). Most informaticians agreed that AI/ML should be independently vetted and standardized prior to use in a clinical setting (96.0%), regulated (95.6%), and evaluated in randomized controlled trials (81.2%).

The three most common reasons respondents identified as barriers to the successful development of AI/ML in healthcare were data quality (67.3%), lack of standardization (39.8%), and difficulty of acceptance by healthcare providers (35.7%).

### Healthcare informatician—attitudes towards AI/ML for the management of blood clots

**Figure 2:**
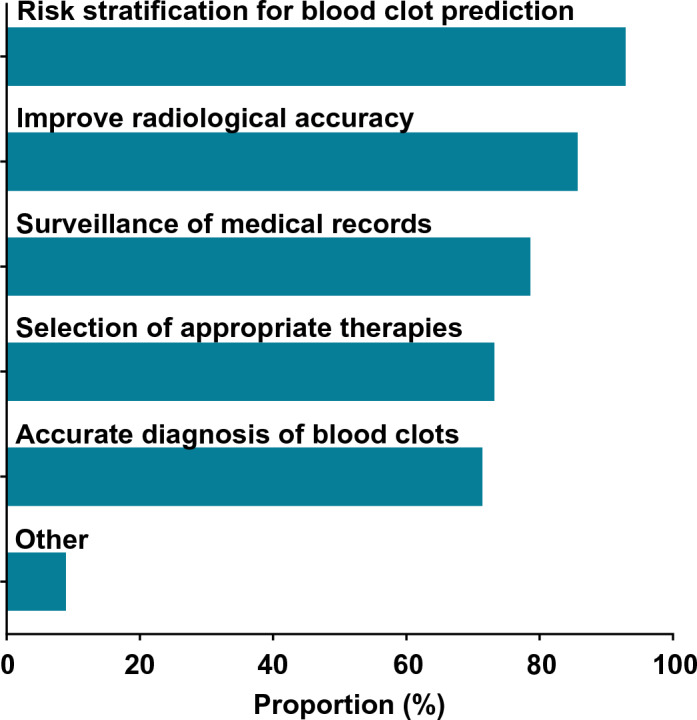
Potential applications of AI/ML for management of blood clots (n = 56).

A majority of informaticians agreed that AI/ML can be used for clinical management of blood clots (56.0% 95% CI 46–66%). Of these 56 respondents, most agreed that AI/ML can be used for risk stratification (94.6%), radiologic accuracy (87.5%), surveillance (80.4%), diagnosis (73.2%), and treatment (73.2%) (Fig. 2). Four respondents proposed potential additional uses for AI/ML: monitoring the process of clot dissolution, warfarin dosing, shared decision-making, and treatment during acute versus chronic recovery phases. All respondents were asked about perceived barriers, and the most commonly cited barriers were a lack of transparency with AI/ML systems (48.5%), concern that clinicians would not use an AI/ML system (34.7%), and concerns around liability (24.8%) (Supplementary Table 2). Informaticians who identified as clinicians were more likely to think that AI can help with VTE compared to those who did not identify as clinicians, though the difference was not statistically significant (66.7% vs. 47.3%, respectively; p = 0.052). Respondents at organizations that have implemented AI were not significantly more likely to think that AI can help with VTE compared to those at organizations that had not implemented AI (59.0% vs. 48.7%; p = 0.32).

All respondents were asked the free-text question, “What else should be considered when using AI/ML to assist in the clinical management of blood clots?” There were 37 responses, six of which were removed from analysis because they did not answer the questions. Of the remaining 31 responses, nearly all were related to validation of the system and discussed factors related to testing, bias, and transparency. Several responses discussed deployment, and a few responses touched on the importance of clinician oversight (themes in Table 3; coding tree in Supplementary Table 3).

**Table 3:** Informatician recommendations regarding AI/ML for management of blood clots.

Informatician concerns around AI/ML for VTE prevention	
Theme	Examples
Validation of the AI/ML system	“Data quality in EHRs is rather poor and can introduce multiple errors. There needs to be extensive vetting and validation checking of AI results and the methods of development must be transparent if it is to be trusted” “The deployment of such a system should be done extremely carefully and new types of trials should be created, where an independent regulating body can assess the performance of said system”
Deployment	"Need to make sure that it fits into the workflow, and it is somewhat context aware” “…deployment considerations (e.g., human-in-the-loop workflow development, and monitoring for drift)”
Importance of clinical judgment	“Keep in mind that the system helps the clinicians and patients, but they have the ultimate choice in what to do” “What human oversight is there for edge cases or ethical oversight?”

*AI* Artificial intelligence, *ML* Machine learning, *EHR* Electronic health record.

## Clinician attitudes towards AI/ML for the management of blood clots

Most respondents (70.1%, 95% CI: 66.6–73.6) believed that AI/ML probably or definitely can help with VTE prevention. Respondents who had used AI in clinical practice were more likely to agree that AI can help (RR: 1.45, 95% CI: 1.33–1.58) compared to non-AI users. Respondent age and physician training status were not associated with the belief that AI can help (both p ≥ 0.05).

Respondents who answered anything other than “definitely yes” to whether AI/ML can help with VTE prevention were asked about their concerns. The three most common concerns were a lack of transparency (25.4%), that AI/ML is not ready for clinical use (25.0%), and that AI/ML is not accurate enough (22.7%). Seventy respondents provided free-text responses. A total of 34 comments were removed (16 responses were removed for comments such as “I don’t have enough knowledge in this area,” 13 responses were removed for comments such as “I have no concerns,” and 5 responses were removed for comments requesting more information about a specific AI/ML system.) The remaining 36 comments were categorized into the following themes: the importance of clinical judgment, concerns around how the AI/ML system is validated, and concerns around deployment of the system and the possibility of technology fatigue (themes in Table 4; coding tree in Supplementary Table 4).

**Table 4:** Clinician concerns around AI/ML for VTE prevention.

Clinician concerns around AI/ML for VTE prevention	
Theme	Examples
Importance of clinical judgment	“Too much trust might be placed in AI, rather than it prompting independent clinical judgment” “For routine VTE situations it might be suitable, but there are too many other situations where clinical judgment is necessary”
Validation of the AI/ML system	“Not ready for clinical use in a small subspecialty population like our hospital. Ready for ordinary adult hospital use” “Lack of studies showing clinical outcomes”
Deployment and technology fatigue	“With the critical staff shortage in all departments, nursing does not have the time available to troubleshoot a new system” “Our EMR is already difficult to use and full of glitches… makes it hard to trust other tools” “I question if our medical system is broadly ‘tech-savvy’ enough. We still fax medical records!”

*VTE* venous thromboembolism, *AI* artificial intelligence, *ML* machine learning, *EMR* electronic medical record.

## Discussion

Although informaticians expressed concern that clinicians would not be willing to use AI/ML, a majority of clinician respondents in our survey believed that AI/ML can be helpful in the field of VTE prevention and management. This is consistent with surveys evaluating clinician attitudes towards AI/ML in other specialties; generally, clinicians have reported that AI/ML will have a positive impact on their field^19–21^. Surveys have found that some specialists may be less likely to perceive benefits from AI/ML^22^. As AI/ML technology advances and becomes more accessible, further research into clinician and other stakeholder views is critical in order to understand how AI/ML can positively and safely impact care.

Both clinicians and informaticians cited transparency as their number one concern when considering AI/ML systems for the management of blood clots. Though transparency has been cited as a barrier in other clinician surveys, it was not the main concern. As AI/ML moves closer to the bedside, concerns around the “black box” nature of AI/ML may increase. In a landmark paper from 2017, Doshi-Velez and Kim discuss how different measures of success have emerged as AI/ML becomes more ubiquitous^23^. Initially, models were optimized for performance, but now there are efforts to optimize models for other important criteria such as safety and nondiscrimination. In order to evaluate for and reinforce these criteria, experts have called for increased *interpretability* of AI/ML. Doshi-Velez and Kim define interpretability as “the ability to explain or to present in understandable terms to a human” and discuss how interpretability can allow humans to learn, ensure the model is safe and ethical, and weigh competing objectives^23^. These factors may be particularly important in the field of medicine, where care needs to be personalized not only to each individual’s health history, but also to their values and preferences. Interpretability may allow clinicians to readily weigh their own clinical judgment, a factor that both clinicians and informaticians identified as important. Efforts are being made to further define what it means for AI/ML systems to be interpretable, and how that can be technically accomplished^24^.

Both informaticians and clinicians discussed the importance of how AI/ML systems are validated and deployed, touching on subjects such as the quality of electronic health record data, whether the system can be applied to patient populations other than which it was trained , how to avoid perpetuating bias, and how the system will fit into the clinical workflow. Organizations are working to define standards for how AI/ML studies report their data and how these models might be evaluated in clinical trials^25,26^. Leaders also recognize the numerous factors that need to be addressed before AI/ML is brought to the bedside including safety, ethical, and security considerations^27–29^, and guidelines are being developed to support healthcare organizations looking to deploy these systems^30^.

When asked about potential concerns around the use of AI/ML in VTE prevention, several clinicians shared that they did not know enough about the field of AI/ML to comment. This self-identified knowledge gap has been voiced in other studies^20^, and some experts argue that clinicians will need to learn more about the field given AI/ML’s inevitable arrival in healthcare^31^. Clinicians will be needed to serve as peer reviewers and clinical trial leaders, as well as to make the ultimate decision as to whether a new system has a clinically relevant impact on their patients^31^. While clinicians cannot be expected to become experts in every aspect of medicine, it is clear that we will need leaders in this area in order to ensure that AI/ML enters healthcare in a safe, clinically effective, and ethical manner.

There are several limitations to our study. We used online platforms such as message boards and social media websites for survey distribution, which allowed for wide dissemination but did not allow for an accurate response rate calculation, which can underestimate or obscure sampling bias. Given participation was voluntary, the respondents who elected to participate may be more comfortable with technology and more likely to view AI/ML positively. The majority of clinician respondents were younger than 40 years old, and while these findings may reflect the views of a new generation of practitioners, they may not reflect the views of older clinicians in leadership roles who have the ability to effect change today. We used broad terms such as "blood clot” and “AI/ML” in our surveys in order to assess general attitudes and account for respondents who may not have a clinical or technical background, respectively. Several respondents commented on the difficulty of assessing these broad fields, and further research will need to be done evaluating specific algorithms in specific clinical contexts. Additionally, further studies evaluating the opinions and preferences of patients will need to be done^21^.

## Conclusion

A majority of clinicians and healthcare informaticians believe AI/ML can help with VTE management. Both groups raised concerns around how the AI/ML system is validated, how the system is ultimately deployed in the clinical setting, and the importance of clinical judgment. Further studies of specific AI/ML systems are needed to understand how they can impact clinical care.

### Supplementary Information

Supplementary Information.

## Data Availability

Data is available upon request; researchers interested in reviewing our data should contact Rushad Patell at rpatell@bidmc.harvard.edu.
