# Supplementary material for: The potential use of artificial intelligence for venous thromboembolism prophylaxis and management: clinician and healthcare informatician perspectives
Source: Sci Rep. 2024 May 26;14:12010. doi: 10.1038/s41598-024-62535-9 (PMC11127994; doi:10.1038/s41598-024-62535-9)
Supplement: Supplementary file 1 — Supplementary Information. [file 41598_2024_62535_MOESM1_ESM.docx]

**Supplementary data 1:** Clinician survey

We are conducting a survey to better understand how clinicians utilize venous thromboembolism (VTE) prophylaxis for hospitalized adult patients. VTE includes deep vein thrombosis (DVT) and pulmonary embolism (PE). Prophylaxis includes mechanical and pharmacologic measures.

Your participation is completely voluntary, anonymous, and will not impact your employment. This survey will take approximately 10 minutes to complete. This survey was approved by IRB Protocol #2021P000471. If you have questions or concerns, please contact us at rpatell@bidmc.harvard.edu or blam@bidmc.harvard.edu. Thank you!

Section 2: Attitudes and practices regarding artificial intelligence in medicine

Artificial intelligence refers to technologies that mimic human intelligence. Machine learning, for example, uses large datasets to “train” a model to predict outcomes or help make decisions.

Have you ever used artificial intelligence to inform your clinical practice?

- Yes
- No
- Not sure

Can artificial intelligence help ensure appropriate VTE prophylaxis for hospitalized patients?

- Definitely yes
- Probably yes
- No opinion
- Probably not
- Definitely not

*If select Anything other than “Definitely yes”*

What concerns do you have about artificial intelligence? Check all that apply:

- Artificial intelligence would be too costly to develop
- I would not trust artificial intelligence
- I do not think artificial intelligence is accurate enough
- There is a lack of transparency with artificial intelligence
- Artificial intelligence is not ready for clinical use
- Other (please explain)

Section 3: Demographics

What is your role?

- MD or DO
- Nurse Practitioner
- Physician Assistant
- Nurse
- Pharmacist
- Other (please specify)

*If select MD or DO, Nurse Practitioner, or Physician Assistant*

Are you still in training (a resident or fellow)?

- Yes
- No

*If select Yes, still in training*

What is your post-graduate year level?

- PGY1
- PGY2
- PGY3
- PGY4
- PGY5
- PGY6
- PGY7 or above

*If select No, not in training*

How long have you been practicing?

- Less than 1 year
- 1-5 years
- 6-10 years
- More than 10 years

What is your focus of clinical practice? (Check all that apply)

- Critical Care
- Hematology
- Hospital Medicine
- Oncology
- Primary Care
- Pulmonary Medicine
- Cardiology
- Vascular Medicine
- Other (please specify)

How would you characterize your primary practice setting?

- Academic Hospital
- VA Hospital
- Community Hospital
- Other (please specify)

Do you practice in the United States?

- Yes
- No

Where do you currently practice?

- North America
- South America
- Europe
- Africa
- Asia
- Australia

Do you do research in thrombosis/hemostasis?

- Yes
- No

Do you have a specific interest in VTE prophylaxis?

- Yes
- No

Do you have a specific interest in informatics?

- Yes
- No

What is your age?

- 20 years or less
- 21 - 30 years old
- 31 - 40 years old
- 41 - 50 years old
- 51 - 60 years old
- 61 years or older

Do you currently describe yourself as female, male, or transgender?

- Female
- Male
- Transgender
- Other

What is your race? Check all that apply.

- American Indian or Pacific Native
- Asian
- Black or African American
- Native Hawaiian or Pacific Islander
- White
- Other (please specify)

What is your ethnicity?

- Hispanic or Latinx
- Non-Hispanic or Latinx
- Other

Thank you for completing our survey! Your responses are very valuable and appreciated!

**Supplementary data 2:** Informatician survey

Blood clots are a frequent and potentially fatal health condition. Many blood clots occur in the setting of other illnesses, hospitalizations or after medical procedures and surgeries. We are conducting a survey to better understand attitudes towards artificial intelligence/machine learning and its potential use in blood clot prevention and surveillance.

As someone who works closely in the field of informatics you are invited to participate and expand our current understanding of the field.

Study participation is voluntary. If you choose to be in the research study, please complete the survey below. The survey will take about 10 minutes for you to complete.

You can skip any survey questions that you do not want to answer. Even if you start the survey, you are not required to complete it, partial responses will be saved. You can stop at any time. The survey is anonymous, and no one will be able to link your answers back to you. Please do not include your name or other information that could be used to identify you in the survey responses.

Being in this study is voluntary. Study was approved by IRB at Beth Israel Deaconess Medical Center (Protocol 2022P000132). Please contact Rushad Patell at rpatell@bidmc.harvard.edu with questions about this research study. If you have questions about your rights participating in research or would like to speak with someone independent from the research team, please contact the Human Subject Protection Office (617) 975-8500.

Thank you for participating!

How do you informed do you believe yourself to be about artificial intelligence/machine learning (AI/ML)?

- Very well informed
- Sufficiently informed
- No opinion
- Poorly informed
- Very poorly informed

What is your exposure to AI/ML? Select all that apply.

- I have taken coursework on the topic
- I have worked on deploying an AI/ML system
- I do research on the topic – domain expert
- I have used it for my business practice
- I have used it for my clinical practice
- I have had no exposure to AI/ML
- Other (please specify)

Does your organization use or develop AI/ML in any capacity for healthcare?

- Yes
- No
- I don’t know

*If Yes,*

Describe the status of AI/ML implementation at your organization for healthcare:

- Implemented, with multiple models in use
- Implemented, with 1-2 models in use
- Not implemented, but resources are allocated
- Not implemented, with no known plans
- Other (please specify)

How does your healthcare organization procure AI/ML products? Select all that apply.

- I do not work directly or indirectly with a healthcare organization
- From 3^rd^ party vendors
- We partner with local universities
- We develop our own products
- Other (please specify)

What are your views on AI/ML?

|  | Definitely not | Probably not | No opinion | Probably yes | Definitely yes |
| --- | --- | --- | --- | --- | --- |
| AI/ML can have a positive impact on the care of patients |  |  |  |  |  |
| AI/ML can help healthcare organizations meet regulatory requirements |  |  |  |  |  |
| Overall, I have found AI/ML to be reliable |  |  |  |  |  |
| AI/ML has the potential to perform better than humans |  |  |  |  |  |
| AI/ML can have a positive economic impact on my healthcare organization |  |  |  |  |  |
| AI/ML will replace human employees in some jobs |  |  |  |  |  |
| I would trust my own care to an AI/ML system |  |  |  |  |  |
| AI/ML should be independently vetted and standardized prior to use in a clinical setting |  |  |  |  |  |
| AI/ML should be regulated in healthcare |  |  |  |  |  |
| I would trust a closed proprietary AI/ML for use in a clinical setting |  |  |  |  |  |
| AI/ML should be evaluated in randomized control trials before they are deployed in clinical settings |  |  |  |  |  |

What about AI/ML and blood clots?

Do you think AI/ML can be used for clinical management of blood clots?

- Yes
- No
- I don’t know

*If Yes,*

What aspects of blood clot management do you think AI/ML could be useful for?

- Risk stratification for blood clot prediction
- Accurate diagnosis of blood clots
- Improve radiological accuracy
- Selection of appropriate therapies
- Surveillance of medical therapies
- Other (please specify)

What barriers do you think exist to using AI/ML for management of blood clots? Select all that apply.

- It would be too difficult to implement AI/ML
- I do not think an AI/ML system would be accurate
- I do not think our clinicians would use an AI/ML system
- I do not think our patients would trust an AI/ML system
- I am worried about liability
- There is a lack of transparency with AI/ML systems
- Other (please specify)

Which are the three major challenges for the successful development of AI/ML in healthcare? Pick up to three.

- Data quality
- Data not formatted for downstream use
- Mostly retrospective data
- Single center / single system data
- Lack of standardization
- Lack of open systems
- Technical (e.g. algorithmic) limitations
- Difficulty of acceptance by healthcare providers
- Difficulty of acceptance by patients
- Ethical considerations (e.g. decisions that can affect patient management)

What else should be considered when using AI/ML to assist in the clinical management of blood clots?

______________________________________________________________________

______________________________________________________________________

Tell us about yourself

I am a (select all that apply):

- CMIO, or other medical informatics officer
- Clinician
- Data scientist
- Clinical informaticist
- Biomedical informaticist/Computational biologist
- Other (please specify)

How long have you been practicing in informatics?

- I do not practice in the informatics field
- Less than 1 year
- 1-5 years
- 6-10 years
- More than 10 years

If you are developing AI/ML systems, please check all that apply:

- I work on natural language processing
- I work on binary classification
- I work on multi-class classification
- I work on regression
- I use supervised learning approaches
- I use unsupervised learning approaches
- I use semi-supervised learning approaches
- I use transfer learning approaches
- Other (please specify)

If you are developing AI/ML systems, please check all that apply:

- I implement in Python
- I implement in R
- I implement in Matlab
- I implement in Julia
- I utilize a GUI-based product
- Package – Toolkit of preference (e.g. scikit, TensorFlow, Caret, H2O, TidyModels, etc.)
- Other (please specify)

How old are you?

- 20 years or less
- 21-30 years old
- 31-40 years old
- 41-50 years old
- 51-60 years old
- 61 years or older

Do you currently describe yourself as female, male, or transgender?

- Female
- Male
- Transgender
- Other
- Prefer not to answer

What is your race? Select all that apply.

- American Indian or Alaska Native
- Asian
- Black or African American
- Native Hawaiian or Pacific Islander
- White
- Other
- Prefer not to answer

What is your ethnicity?

- Hispanic or Latino
- Not Hispanic or Latino
- Other
- Prefer not to answer

Would you be interested in participating in a focus group to help us better understand how informaticists think about artificial intelligence?

- Yes
- No

**Supplementary Table 1:** Informatician attitudes towards AI/ML (n=101)

|  | **Definitely not** | **Probably not** | **No opinion** | **Probably yes** | **Definitely yes** |
| --- | --- | --- | --- | --- | --- |
| **AI/ML can have a positive impact on the care of patients** | 0 (0.0%) | 2 (2.0%) | 3 (3.0%) | 43 (42.6%) | 53 (52.5%) |
| **AI/ML can help healthcare organizations meet regulatory requirements** | 0 (0.0%) | 2 (2.0%) | 13 (13.0%) | 53 (53.0%) | 32 (32.0%) |
| **AI/ML has the potential to perform better than humans** | 1 (1.0%) | 6 (5.9%) | 17 (16.8%) | 43 (42.6%) | 34 (33.7%) |
| **AI/ML can have a positive economic impact on my healthcare organization** | 0 (0.0%) | 6 (6.0%) | 13 (13.0%) | 49 (49.0%) | 32 (32.0%) |
| **AI/ML will replace human employees in some jobs** | 5 (5.0%) | 22 (21.8%) | 13 (12.9%) | 38 (37.6%) | 23 (22.8%) |
| **I would trust my own care to an AI/ML system** | 10 (9.9%) | 19 (18.8%) | 22 (21.8%) | 39 (38.6%) | 11 (10.9%) |
| **Overall, I have found AI/ML to be reliable** | 0 (0.0%) | 15 (14.9%) | 27 (26.7%) | 45 (44.6%) | 14 (13.9%) |
| **AI/ML should be independently vetted and standardized prior to use in a clinical setting** | 0 (0.0%) | 1 (1.0%) | 3 (3.0%) | 16 (15.8%) | 81 (80.2%) |
| **AI/ML should be regulated in healthcare** | 0 (0.0%) | 2 (2.0%) | 3 (3.0%) | 23 (22.8%) | 73 (72.8%) |
| **I would trust a closed proprietary AI/ML for use in a clinical setting** | 17 (16.8%) | 23 (22.8%) | 21 (20.8%) | 35 (34.7%) | 5 (5.0%) |
| **AI/ML should be evaluated in randomized control trials before they are deployed in clinical settings** | 1 (1.0%) | 12 (11.9%) | 6 (5.9%) | 25 (24.8%) | 57 (56.4%) |

AI = artificial intelligence; ML = machine learning

**Supplementary Table 2:** What barriers do you think exist to using AI/ML for management of blood clots?

| **Barriers to AI/ML for management of blood clots** | **N=101*** |
| --- | --- |
| There is a lack of transparency with AI/ML systems | 49 (48.5%) |
| I do not think our clinicians would use an AI/ML system | 35 (34.7%) |
| I am worried about liability | 25 (24.8%) |
| I do not think our patients would trust an AI/ML system | 21 (20.8%) |
| It would be too difficult to implement AI/ML | 13 (12.9%) |
| I do not think an AI/ML system would be accurate | 11 (10.9%) |
| Other | 24 (23.8%) |

*Sum may be greater than the total because participants could select multiple responses
AI = artificial intelligence; ML = machine learning

**Supplementary Table 3:** Coding tree for free-text question for informaticians, “What else should be considered when using AI/ML to assist in the clinical management of blood clots?”

| **Themes** | **Subthemes** |
| --- | --- |
| Validation of the AI/ML system | Accuracy   - Demonstration of accuracy - Ability to appropriately weigh risk factors - Accuracy of underlying data   Validation   - Demonstration of effectiveness - Testing prior to deployment |
| Deployment | Technology fatigue   - Reluctance to add features to the electronic health record - Alert fatigue - Concerns around increased workflow burden   Staffing needs   - Need for staff education - Availability of staff for troubleshooting technology - Staff acceptance of new technology |
| Importance of clinical judgment | Overreliance   - Clinicians will lose insight into risk stratification - Clinicians will not question the AI system   Clinical judgment is superior   - Clinicians can better weigh other risk factors - Clinicians can better manage rare scenarios - Clinicians can better personalize decisions to the patient |

AI = artificial intelligence; ML = machine learning

**Supplementary Table 4:** Coding tree for free-text question for clinicians “What concerns do you have about AI?”

| **Themes** | **Subthemes** |
| --- | --- |
| Importance of clinical judgment | Clinical judgment is superior   - AI may not catch everything - Clinicians can better personalize decisions to the patient - Clinicians can better weigh other risk factors   AI is a supplement and not a replacement   - Clinicians may come become overly reliant on AI - Humans should make final treatment decisions |
| Validation of the AI/ML system | Readiness for clinical use   - Need more evidence for safety - Need proof of benefit - Need to show accuracy in diverse clinical scenarios   Lack of trust   - Staff comfort with new technology - Concern around biases - Reliability of underlying data |
| Deployment and technology fatigue | Technology fatigue   - Lack of trust in technology in general - Concerns around increased workload - Alert fatigue   Cost   - High cost of development - High cost of deployment |

AI = artificial intelligence; ML = machine learning
